# The impact of the introduction of selective screening in the UK on the epidemiology, presentation, and treatment outcomes of developmental dysplasia of the hip

**DOI:** 10.1302/2633-1462.48.BJO-2022-0158.R1

**Published:** 2023-08-23

**Authors:** Arwel T. Poacher, Isaac Hathaway, Daniel L. Crook, Joseph L. J. Froud, Lily Scourfield, Catherine James, Matthew Horner, Eleanor C. Carpenter

**Affiliations:** 1 Trauma Department, University Hospital of Wales, Cardiff, UK; 2 Cardiff University School of Medicine, Cardiff, UK; 3 Department of Surgery, Royal London Hospital, London, UK; 4 Guy's and St Thomas' NHS Foundation Trust, London, UK; 5 Noah’s Ark Children’s Hospital for Wales, Cardiff, UK

**Keywords:** Developmental dysplasia of the hip, Universal screening, Selective screening, Paediatrics, Paediatric hip, Developmental dysplasia of the hip (DDH), epidemiology, Physical Examination, morbidity, MEDLINE, hips, surgical treatment, dislocation of the hip, hip deformity, torticollis

## Abstract

**Aims:**

Developmental dysplasia of the hip (DDH) can be managed effectively with non-surgical interventions when diagnosed early. However, the likelihood of surgical intervention increases with a late presentation. Therefore, an effective screening programme is essential to prevent late diagnosis and reduce surgical morbidity in the population.

**Methods:**

We conducted a systematic review and meta-analysis of the epidemiological literature from the last 25 years in the UK. Articles were selected from databases searches using MEDLINE, EMBASE, OVID, and Cochrane; 13 papers met the inclusion criteria.

**Results:**

The incidence of DDH within the UK over the last 25 years is 7.3/1,000 live births with females making up 86% of the DDH population (odds ratio 6.14 (95% confidence interval 3.3 to 11.5); p < 0.001). The incidence of DDH significantly increased following the change in the Newborn and Infant Physical Examination (NIPE) guidance from 6.5/1,000 to 9.4/1,000 live births (p < 0.001). The rate of late presentation also increased following the changes to the NIPE guidance, rising from 0.7/1,000 to 1.2/1,000 live births (p < 0.001). However, despite this increase in late-presenting cases, there was no change in the rates of surgical intervention (0.8/1,000 live births; p = 0.940).

**Conclusion:**

The literature demonstrates that the implementation of a selective screening programme increased the incidence of DDH diagnosis in the UK while subsequently increasing the rates of late presentation and failing in its goal of reducing the rates of surgical intervention for DDH.

Cite this article: *Bone Jt Open* 2023;4(8):635–642.

## Introduction

Developmental dysplasia of the hip (DDH) encompasses a wide spectrum of conditions, from subtle acetabular dysplasia to irreducible dislocation of the hip.^1^ DDH is a common cause of childhood disability and is the primary cause of premature arthritis in the young and the leading cause of hip arthroplasty under the age of 60.^2,3^ Furthermore, early diagnosis of DDH is a crucial factor in reducing the need for surgical intervention and the incidence of disability in childhood and later life.^4^ Therefore, effective screening of DDH is essential in improving paediatric public health and reducing the burden on the health service.^5^

Various screening programmes have been established within countries with the appropriate infrastructure to try and ensure early diagnosis of DDH.^6^ Early diagnosis allows the compliant soft-tissues around the neonatal hip to be manipulated more effectively, and the immature acetabulum physiologically remodelled without the need for surgical intervention.^7,8^ Early detection is most commonly defined as < 12 weeks of chronological age,^9^ and late detection is commonly described as > 12 weeks. Most institutions use conservative treatment with an abduction brace such as the Pavlik harness, and achieve high rates of success in those who are detected early.^10^ However, the success of bracing therapy decreases with age,^11^ and therefore many late-presenting or missed cases will require surgical intervention in the form of closed or open reductions. Older children may also require bony acetabular and/or femoral osteotomies to fully correct the hip deformity, and reduce the long-term disease-related morbidity.^8^ These invasive surgical procedures come with significant morbidity and mortality: for example, a positive correlation between age of DDH diagnosis and severity of avascular necrosis (AVN) has been well established.^12,13^

The poor outcomes of late-presenting DDH highlight the importance of effective early detection of the disease.^14-17^ However, clinical examination of DDH, including the Ortolani and Barlow manoeuvres and the ‘clicky hip’, demonstrate notoriously poor specificity and sensitivity for detection of both dysplasia and dislocated hips.^8^ The 2008 change screening guidelines for DDH within the UK advise that the newborn and six- to eight-week clinical assessment should be used in combination with ‘selective’ risk factor screening for investigation of at-risk patients using ultrasound,^18,19^ where radiological measurements are used to denote disease severity.^20,21^ The Newborn and Infant Physical Examination (NIPE) guidelines determine that first-degree family history, breech presentation at or after 36 weeks of pregnancy, and all infants from multiple pregnancies, where one of these risk factors is present, warrant an invitation to ultrasound investigation (Table I). However, since the introduction of the NIPE guidelines on DDH screening, there has been little evidence regarding the true impact of these changes on the rates of DDH diagnosis, late-presenting DDH, and the need for surgical intervention.

**Table I.** A table demonstrating the breakdown of different classification of UK screening programmes for developmental dysplasia of the hip.

Type of screening programme	Criteria for invitation to USS investigation	Breakdown of risk factors included	NIPE guidance^16^
Traditional	Abnormal Ortolani or Barlow examination; clicky hips; limited abduction etc.	Positive examination finding	No longer accepted as good practice due to poor sensitivity and specificity of examination
Selective	Abnormal exam and/or presence of a primary RF	Breech birth; family history; multiple births (if the sibling has one or more of the above primary risk factors)	Endorsed by NIPE and undertaken routinely across in NHS England
Universal	All new-borns receive USS investigation		Not endorsed in the UK but used in mainland Europe

CTEV, congenital talipes calcaneovalgus; FCEV, fixed congenital talipes equinovarus; NIPE, New-born and Infant Physical Examination; PTEV, postural talipes equinovarus deformity; RF, risk factor; USS, ultrasound.

Therefore, this paper presents the only meta-analysis-derived estimates of the effect of changes to the NIPE guidelines on the incidence of DDH diagnosis, late presentations, and rates of surgical treatment.

## Methods

We performed a comprehensive literature search to identify studies that involved screening for patients with DDH, with a particular interest in late diagnoses. MEDLINE, Embase, OVID, and Cochrane were searched, in addition to a manual search of study citations. Our search terms for both engines were as follows: (((DDH) OR (Developmental Dysplasia of the Hip)) OR (Developmental Dislocation of the Hip)) AND ((Late) OR (Delayed) OR (Missed)), including MeSH terms. Our manual search covered the reference lists of several relevant papers and Cochrane reviews, and involved a separate search on the search engines mentioned above. We considered all papers that involved studies describing diagnostic processes for DDH. We specifically chose to review papers, in lieu of administrative health data, as this would have required ethical approval and may have resulted in less generalizability given limitations related to access. The use of studies was deemed sufficient to highlight any significant results that warrant any future investigation; an empirical or prospective cohort study may be conducted later. No studies were deemed to be overlapping.

Our exclusion criteria were studies that had no reference to DDH, were not written in English, not based in Ireland or the UK, or did not include the basic information (outlined in Table II) needed for the meta-analysis. Our authors reviewed all abstracts independently before jointly deciding which papers matched the relevant inclusion criteria. We included a specific timeframe to ensure we gained a similar number of papers for before and after the introduction of the NIPE guidelines. We sourced papers from 1994 to 2008 (pre-NIPE) and 2008 to 2021 (post-NIPE). Papers were categorized depending on the date at which their protocol was written.

**Table II.** Inclusion/exclusion criteria of the review.

Criteria	Inclusion	Exclusion
Research participant	Research relating to and/or reporting on the epidemiology, presentation, and outcomes of DDH in the UK	Research relating to and/or reporting on the epidemiology, presentation and outcomes of DDH in the UK
Location	Studies performed or reporting on population inside of the UK and Ireland	Studies performed on populations outside the UK
Type of study	Primary research included but not limited to cohort studies, case-control studies, prospective registry studies, epidemiological evaluations, and service evaluations that report findings relevant to the research question whether or not they included an intervention or change	Studies that did not report on the epidemiology of DDH
Methodology	Research involving quantitative, qualitative, or mixed methodology	Commentaries, editorial comments
Timescale	Research published between 1994 and 2021	Research not published between 1994 and 2021

DDH, developmental dysplasia of the hip.

Papers were screened by ATP, IH, and DLC for their suitability for inclusion and any disagreements were settled by a majority decision. Data extracted were name, year, and author(s) of the paper, methods, results, strengths, and weaknesses, in addition to the screening criteria, which is the focus of this review. The numbers of live births, cases of DDH, late presentations, and surgical treatment (all as classified by the authors of the original studies) were the data extracted for use in the meta-analysis. Late presentation was taken as those presenting over 12 weeks as this was the majority criterion among the publications. DDH was classified by each study and in those included, it was defined as Graf classification II to IV. Surgical intervention was defined as any closed or open intervention of the DDH-affected hip. Meta-analysis was performed in R v. 4.0.5 “Shake and Throw” (R Foundation for Statistical Computing, Austria), using standard DerSimonian and Laird meta-analysis models with conventional measures of heterogeneity and levels of significance chosen. Data and the code for the models used are available upon request.

## Results

**Fig. 1:**
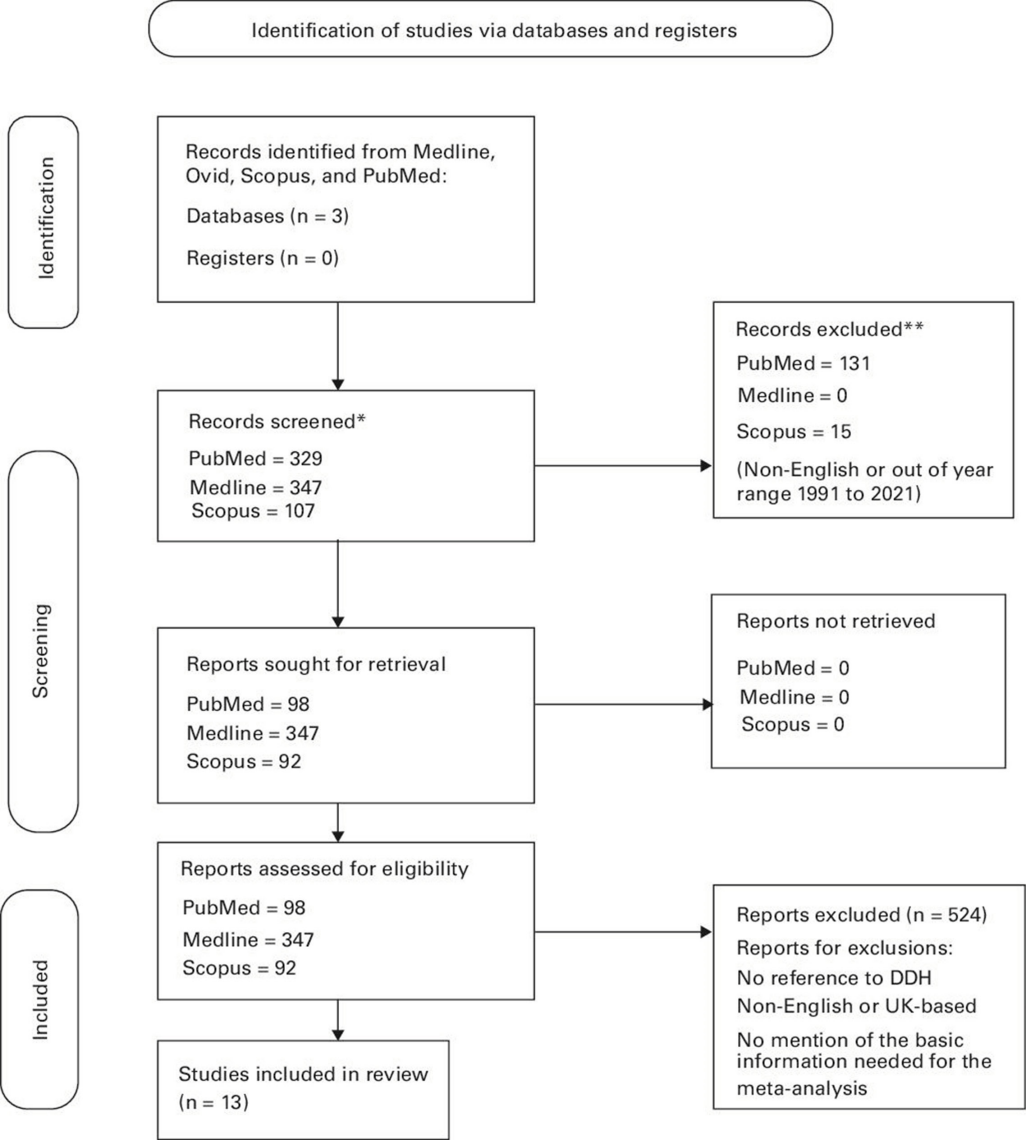
PRISMA 2020 flow diagram for new systematic reviews which included searches of databases and registers only. *Consider, if feasible to do so, reporting the number of records identified from each database or register searched (rather than the total number across all databases/registers).**If automation tools were used, indicate how many records were excluded by a human and how many were excluded by automation tools. DDH, developmental dysplasia of the hip.

For the 1994 to 2008 search, PubMed yielded a total of 76 citations, of which 68 were excluded, while MEDLINE yielded seven citations, of which six were excluded. For the 2008 to 2021 search, PubMed yielded a total of 158 citations, of which 155 were excluded, while MEDLINE yielded 30 citations, of which 29 were excluded (Figure 1). Automation tools were not used during the data search and extraction.

Our search yielded 271 studies, of which 13 were selected for full assessment based on our inclusion criteria. The incidence of late-presenting DDH was recorded in 12 studies and surgical management was recorded by nine studies (Supplementary Table i).^22^ Of the studies, 13 contained the required data for inclusion in the meta-analyses. The summary characteristics of these studies are in (Supplementary Table i). The rate of non-surgical treatment was not addressed in this review due to lack of useable data within the literature.

### Meta-analysis

The incidence of DDH is poorly defined within a UK population, and incidence is often referenced from decades-old studies,^23,24^ or literature that have populations not specific to the UK. Therefore, we have summarized all studies completed within the UK and Ireland (Table III, Supplementary Material) that contained the data required for an analysis of the incidence of DDH. A fixed-effects meta-analysis (FEM) (Table III) gave the pooled estimate for incidence of DDH of 7.3 cases of DDH per thousand live births (95% confidence interval (CI) 7.1 to 7.6); prior to the implementation of this NIPE guidance (pre-2008), the rate of DDH in the UK population was 6.5 per thousand live births (95% CI 6.2 to 6.8). After the 2008 change to the NIPE guidance, the incidence was estimated as 9.4 per thousand live births (95% CI 8.9 to 10), a significant increase from pre-change levels (p < 0.001). Examination of the sex ratio in our population demonstrated that females are affected by DDH at much higher rates than males. Within our model, females made up 86% of all DDH cases in the UK with an odds ratio of 6.14 (95% CI 3.3 to 11.5; p < 0.001).

**Table III.** Tabulated results of the meta-analyses, data given as the point estimate with 95% confidence intervals. Fixed-effects models analysis results before and after the inclusion of the change in guidance have been included, presenting the ‘pooled’ (1994 to 2021), pre-change (1994 to 2008), and post-change (2009 to 2021), incidence of the late-presenting and surgically treated developmental dysplasia of the hip.

Variable	Rate per 1,000 live births (95% CI)	p-value
Pooled incidence	7.3 (7.1 to 7.6)	
Pre-change	6.5 (6.2 to 6.8)	
Post-change	9.4 (8.9 to 10)	< 0.001
Pooled late presentations	0.8 (0.7 to 0.9)	
Pre-change	0.7 (0.6 to 0.8)	
Post-change	1.2 (1.0 to 1.4)	< 0.001
Pooled surgically treated	0.8 (0.8 to 0.9)	
Pre-change	0.8 (0.8 to 0.9)	
Post-change	0.8 (0.7 to 1.1)	0.980

CI, confidence interval.

The pooled rate of late-presenting cases (defined as > 12 weeks of age) in the UK over the last 25 years was 0.8 (95% CI 0.7 to 0.9) per thousand live births. The model demonstrated a significant increase in the numbers of those presenting late following the implementation of the 2008 NIPE guidance change, rising from 0.7 (95% CI 0.6 to 0.8) per thousand live births to 1.2 (95% CI 1.0 to 1.4) per thousand live births (p < 0.001). However, there was no significant change in the rates of surgical intervention, with our data demonstrating an unchanged rate of 0.8 (95% CI 0.8 to 0.9) per thousand live births pre-change and 0.8 (95% CI 0.7 to 1.1) per thousand live births post-change (p = 0.980), and a pooled rate of 0.8 (95% CI 0.8 to 0.9) per thousand live births. The significant increase in the rates of late presentations without the subsequent expected increase in the rates of surgical intervention suggests that the relationship of surgical intervention with late-presenting cases is not as strong as traditionally thought.

There was a large degree of heterogeneity in all the models. However, as the characteristics estimated are those for which there is one natural value, rather than a range of effect sizes, the fixed-effect method of estimation is likely the most appropriate measure. Heterogeneity was thought to result mainly from the variation in the populations in whom the original studies were conducted, and from some differences in the classifications of DDH — notably, how late presentations were classified varied between papers. We evaluated the overall risk of bias in the studies collected as moderate. Bias was further investigated with standard funnel plots and deemed acceptable. We felt that our search strategy was suitable and returned – as far as we can determine – all the relevant studies in the main medical journal databases. Further, we felt that the large size of the studies included was itself protective from publication bias. Forest plot representation of this study’s meta-analysis can be viewed in Supplementary Figures a to e.

## Discussion

The findings of this study provide a novel overview of the epidemiology and management of DDH in the UK. The best-pooled estimate of the rate of DDH was 7.3 per thousand live births, which is concordant with the range of five to 30 per thousand live births referenced by most studies in the recent literature,^25,26^ and our results also reflected and narrowed down this wide range in a selectively screened population. The model estimates indicate there is a significantly higher incidence of DDH following the 2008 change to the NIPE guidance, and this agrees with the results from a 2016 study of incidence of DDH in a selective screening population of 5.0 per thousand live births.^27^

The mean late diagnosis rate from the results of our meta-analysis, before the introduction of NIPE guidelines, was 0.7 per thousand live births. However, following the introduction of selective screening, the late diagnosis rate increased significantly to 1.2 per thousand live births. However, despite an increase in the rate of late diagnoses, there was no significant effect on the rates of surgical intervention after the introduction of a national selective screening programme. The lack of clarity of the definition of a ‘late’ presentation for diagnostic purposes may have influenced this. System pressures inevitably cause many children to present for initial screening over the age of 12 weeks, however not by a significant amount of time. It is not unreasonable to assume the difference success rates of conservative interventions between those presenting at 12 and 14 weeks are not significant and therefore it may skew our understanding of the severity of ‘late’ presenting cases including these cases in an analysis of late presenters. However, once children reach six months of age their likelihood of conservative management failure is dramatically increased.^28^ Including these in the same group of ‘late’ presenters may be hindering our understanding of DDH and its management. Therefore, we suggest that definitive terminology relating to the age of presentation is adhered to in future literature, for example, the clarification and stratification of a late case of DDH (> 12 weeks) and a missed case of DDH (> 24 weeks). The most effective definition of a missed case is yet to be correlated clinically — for instance, the seminal paper by Broadhurst et al^23^ describes the limitations of clinical coding categories whereby codes only exist in categories of year groups. Therefore, the implementation of a national coding of late and missed cases of DDH would be of significant value when evaluating the impact of these clinical presentations.

Both prior to and following the introduction of NIPE guidelines, most centres used the Graf^20^ grading system of DDH, screening with an examination at the neonatal stage and an examination between six and ten weeks of age. Several centres reported satisfaction with the guidelines and Humphry et al reported improvements in guideline adherence following the introduction of NIPE 2008. However, Reidy et al^29^ expressed concern over the reliability of the presumed safety net of the clinical examination not being reliable, and Phelan et al^16^ went on to emphasize the need for a more robust and regimented screening programme. Whether it is the terms of the guidelines themselves or clinical adherence to them which is preventing a reduction in the number of late diagnoses, there is a pressing need for the literature to examine the true clinical impact of Graf type 2 disease, and to define the progression to pathological disease so that we can effectively evaluate DDH screening.

The current guidance in the UK as advised by NIPE invites those with a breech presentation and first-degree family history to ultrasound screening, alongside those with an abnormal examination. This is based on current evidence, much of which is presented in this review, that demonstrates the efficacy of these ‘primary’ risk factors as predictors of DDH. However, while the relationship of secondary risk factors with DDH has been explored by several studies in this review, due to variation in the definitions of DDH it is difficult to conclude whether these secondary risk factors reduced the rates of late/missed presenting DDH and the need for surgical intervention in a UK population. We should consider evaluating these secondary risk factors within a UK population to assess their efficacy in reducing DDH-related morbidity within our population, in the absence of a universal screening programme.^30^ Studies from mainland Europe have established a significant relationship between DDH and foot deformities,^31-33^ however other studies have not identified a significant association.^34^ Oligohydramnios has been well associated with DDH,^35-37^ but debate continues as to its efficacy as a predictor of DDH.^38,39^ Furthermore, a relationship between torticollis and DDH is well established,^27,34,40-47^ but previous studies have followed small numbers of patients with confirmed torticollis over varying time periods to assess for DDH, which does not truly evaluate its inclusion within a national screening programme. Therefore, further research with consistent classifications of DDH incidence, late presentation, and the need for surgical intervention is essential to establish the association and predictive value of these risk factors within a UK population to assess their efficacy in the context of the NIPE guidance.

Most centres report that they are following the NIPE guidelines accurately, including the key implementation of offering ultrasonography to those infants with positive examination findings or risk factors (all included breech presentation, family history, and multiple pregnancies). However, despite this reported change in practice in accordance with guidance, outcome improvements in the form of improved rates of early DDH detection, with reduced late presentation and reduced rate of surgical intervention, are not demonstrated by the literature. We can speculate on the cause of this, but it is likely multifactorial and context-specific (for example: timing of ultrasound, diagnostic methodology (Graf or other), and human factors associated with the healthcare professional carrying out the neonatal exam). It is also worth noting that the six- to eight-week examination often occurs in primary care, so communication and logistical delays between primary and secondary care, especially following the implementation of a selective screening programme, may account for delays in imaging and diagnosis. Further study into clinical implications of dysplastic (Graf type 2) hips, the treatment outcomes for late compared to missed diagnosis, and the efficacy of secondary risk factors as predictors of disease are required to improve our understanding of DDH, and more effectively evaluate screening programmes in the UK.

One limitation of this study is the use of longitudinal data before and after the engagement of the implementation of the selective screening programme. These data are valuable; however, they must be interpreted in the context of evaluation of change in practice. Most significantly, there has been a general trend away from using surgical intervention in the neonatal population, and therefore rates of surgical intervention may have reduced even without the corresponding reduction in the number of more severe, late-presenting cases of DDH. However, the rate of surgical intervention was unchanged throughout the 25-year time period we studied, which supports the finding that the UK’s selective screening programme has been inadequate in reducing the rates of late presentation and the need for surgical intervention. National surveillance measures with a central database could be used to counteract these limitations. Furthermore, agreement of a universal definition of DDH would provide much-needed clarity to future literature. The use of a universally accepted definition and presentation of outcome associated with Graf grade of DDH will provide significant value for risk stratification and understanding of outcomes from varying severities of DDH, which are not currently well covered.

In conclusion, the 2008 change to NIPE guidance significantly increased the incidence of DDH diagnosis in the UK. However, the implementation of this new screening programme has failed in its goal of reducing the rates of surgical intervention within the neonatal population. Furthermore, the increase in the number of late presentations has not led to an increase in rates of surgical intervention; clearer definitions of a late or missed diagnosis of DDH are required to allow effective evaluation of DDH screening in the UK and abroad.

**Take home message**

- The UK's Newborn and Infant Physical Examination guidelines (NIPE) guidelines for developmental dysplasia of the hip (DDH) advise that the newborn and six- to eight-week clinical assessment should be used in combination with ‘selective’ risk factor screening for investigation of at-risk patients using ultrasound. However, the impact of these changes on the rates of DDH, late presenting DDH, and the need for surgical intervention is poorly understood.

- The evidence presented by this review suggests that the selective screening guidance has been inadequate at reducing the rates of late presentation and need for surgical intervention of DDH. Furthermore, the evidence suggests a need to re-define terminology and compartmentalising the late case (> 12 weeks) and ‘missed’ case (> 24 weeks) in future work relating to screening of DDH.

- Further research evaluating alternative screening strategies, should be undertaken to improve our understanding of the disease burden caused by delayed diagnoses.
